# Sustainability Evaluation of Immobilized Acid-Adapted Microalgal Technology in Acid Mine Drainage Remediation following Emergy and Carbon Footprint Analysis

**DOI:** 10.3390/molecules27031015

**Published:** 2022-02-02

**Authors:** Kuppan Praveen, Sudharsanam Abinandan, Kadiyala Venkateswarlu, Mallavarapu Megharaj

**Affiliations:** 1 Global Centre for Environmental Remediation (GCER), ATC Building, College of Engineering Science and Environment, University of Newcastle, Callaghan, NSW 2308, Australia; Praveen.Kuppan@uon.edu.au (K.P.); abinandan.sudharsanam@newcastle.edu.au (S.A.); 2 Cooperative Research Centre for Contamination Assessment and Remediation of Environment (CRC CARE), ATC Building, University of Newcastle, Callaghan, NSW 2308, Australia; 3 Formerly Department of Microbiology, Sri Krishnadevaraya University, Anantapuramu 515003, India; venkateswarlu.kadiyala@gmail.com

**Keywords:** immobilized microalgae, AMD bioremediation, sustainability, emergy, carbon footprint, NGER, IPCC

## Abstract

Sustainability evaluation of wastewater treatment helps to reduce greenhouse gas emissions, as it emphasizes the development of green technologies and optimum resource use rather than the end-of-pipe treatment. The conventional approaches for treating acid mine drainages (AMDs) are efficient; however, they need enormous amounts of energy, making them less sustainable and causing greater environmental concern. We recently demonstrated the potential of immobilized acid-adapted microalgal technology for AMD remediation. Here, this novel approach has been evaluated following emergy and carbon footprint analysis for its sustainability in AMD treatment. Our results showed that imported energy inputs contributed significantly (>90%) to the overall emergy and were much lower than in passive and active treatment systems. The microalgal treatment required 2–15 times more renewable inputs than the other two treatment systems. Additionally, the emergy indices indicated higher environmental loading ratio and lower per cent renewability, suggesting the need for adequate renewable inputs in the immobilized microalgal system. The emergy yield ratio for biodiesel production from the microalgal biomass after AMD treatment was >1.0, which indicates a better emergy return on total emergy spent. Based on greenhouse gas emissions, carbon footprint analysis (CFA), was performed using default emission factors, in accordance with the IPCC standards and the National Greenhouse Energy Reporting (NGER) program of Australia. Interestingly, CFA of acid-adapted microalgal technology revealed significant greenhouse gas emissions due to usage of various construction materials as per IPCC, while SCOPE 2 emissions from purchased electricity were evident as per NGER. Our findings indicate that the immobilized microalgal technology is highly sustainable in AMD treatment, and its potential could be realized further by including solar energy into the overall treatment system.

## 1. Introduction

Acid mine drainage (AMD), caused by the oxidation of iron sulfide minerals, results in acidic, sulfate-rich water with a low pH and enhanced metal bioavailability that may have serious health and environmental consequences [1]. AMD forms from mining waste rocks, tailings, and mine pits, and it has a wide range of chemical properties, posing a major challenge to the treatment process [2,3]. The efficacy of treatment processes including precipitation, adsorption, electrochemistry, and membrane filtration is influenced by low pH and the presence of high metal concentrations [4]. Chemicals used in the active treatment system of AMD include limestone, hydrated lime, soda ash, caustic soda, ammonia, calcium peroxide, kiln dust, and fly ash [2]. The selection of an AMD treatment approach has traditionally been guided by technical and economic reasons, with environmental performance being a secondary consideration. The long-term durability of remediation systems, on the other hand, is becoming more important in making clear recommendations [5]. As a result, significant efforts have been made to promote an energy-efficient and ecologically friendly wastewater treatment system for AMD [6]. In addition to pollutant removal and achieving the effluent discharge requirement, environmental consequences of chemical and energy consumption, and greenhouse gas (GHG) emissions created by the wastewater treatment process attracted more attention from the environmental scientists [7]. Unlike traditional treatment methods, biological treatments, such as microalgae-based, process utilize CO_2_, wastewater nutrients and sunlight for growth, and present an economic potential for value-added products from algal biomass [8,9,10]. Microalgal technology not only aids in wastewater treatment, but also provides significant environmental, economic, and social advantages by reducing chemical consumption and net pollutant emissions [11,12,13,14].

Life cycle assessment (LCA) is an integrated approach for evaluating the environmental implications of a product or process as well as factors that are undermined in more conventional treatments such as raw material extraction, material transit, and final product disposal [15,16]. Furthermore, only limited investigations have shown the LCA analysis for AMD treatment method [5,17]. Although LCA is a valuable tool for measuring environmental effect, it does not consider the free ecosystem input of the process. Moreover, the amount of natural resource renewability and the planet’s ability to absorb the effects of the manufacturing process have an impact on environmental sustainability and, as a result, repeatability through time [18]. Any approach that considers both the environmental and sustainability aspects of energy systems coupled with the thermos-economic evaluation might be a good substitute. Traditional energy analysis and economic approaches as well as ecological inputs and services have little use in the creation of commercial viability. Reporting for nature’s input is critical for a quantitative knowledge of the interaction between the production system and the biosphere, which is critical for sustainability analysis. Researchers have employed thermodynamic concepts such as matter, energy, and entropy as well as net energy, material yield, and environmental loading to analyze sustainability.

To address the inadequacies of treatment systems, Odum [19] applied emergy analysis, which differentiates free resources (renewable and non-renewable) from those that are acquired. Consequently, a set of emergy indicators and ratios are used to examine the resources from various categories and their influence on emissions. Embodied energy analysis (EEA), which calculates the necessary commercial energy (only includes fossil energy) to generate goods or services, and exergy analysis, which offers only the maximum theoretical work of process in each context, are the two examples of emergy analysis. However, turning all input streams into emergy has a larger boundary and is deemed more complete to measure the ecological cost and relative environmental loss of a system [20]. This feature distinguishes emergy as a compelling alternative for assessing sustainability, which aids decision-making on energy, environmental, and social challenges [21]. Such advancements are most likely to aid in the adoption of industrial practices, allowing for sustainable growth and, as a result, resource conservation in the future. Emergy has been utilized effectively on a variety of systems on many scales in ecological and economic value [22]. Bjorklund et al. [23] conducted an emergy study for a sludge digestion-based integrated wastewater treatment and energy generating system and claimed that wastewater has high emergy content and justified the use of various resources in the treatment process. Zhang et al. [24] also used emergy analysis in scenarios including sewage treatment, treated water discharge, and sludge management for environmental pressure and economic performance. Similarly, CFA accounts for the direct and indirect CO_2_ emissions for each kind of material (concrete, fuel, etc.) or service (material delivery, energy, etc.) used in a treatment system [25,26,27]. Even though CFA methodologies vary, consultants often employ a cross approach that considers both the inputs and outputs of a manufacturing process throughout the development and operation. Similar to emergy analysis, CFA makes comparisons using a single unit and the mass of CO_2_ equivalents (CO_2_e). This implies that both the methods are complimentary as the emergy analysis takes an “upstream” approach, evaluating a system based on the quantity of solar emjoules needed, while CFA is often a “downstream” method. In all, the environmental effect of a system’s operations is analyzed, which can contribute to more robust and sustainable accounting systems [26,27].

Recently, we used acid-adapted microalgal strains, *Desmodesmus* sp. MAS1 and *Heterochlorella* sp. MAS3, to evaluate the immobilized technology for treating synthetic acid mine drainage and demonstrated significant biomass production and iron recovery [4]. Moreover, using LCA to examine the environmental sustainability of the immobilized technology in AMD treatment, we observed minimal energy usage and low emission of GHG as compared to the traditional and hybrid treatment techniques [25]. In the present unique study, we used emergy and carbon footprint analysis to evaluate the immobilized acid-adapted microalgal technology as an effective approach for AMD bioremediation. In fact, the Federal Government of Australia mandated, through NGER system, the threshold criteria for greenhouse gas emissions [28]. Therefore, in the current research, extra analysis was carried out in accordance with the Australian National Greenhouse Gas Reporting Systems to cross-validate the CFA. 

## 2. Results and Discussion

### 2.1. Emergy Flow in Immobilized Acid-Adapted Microalgal System

**Figure 1:**
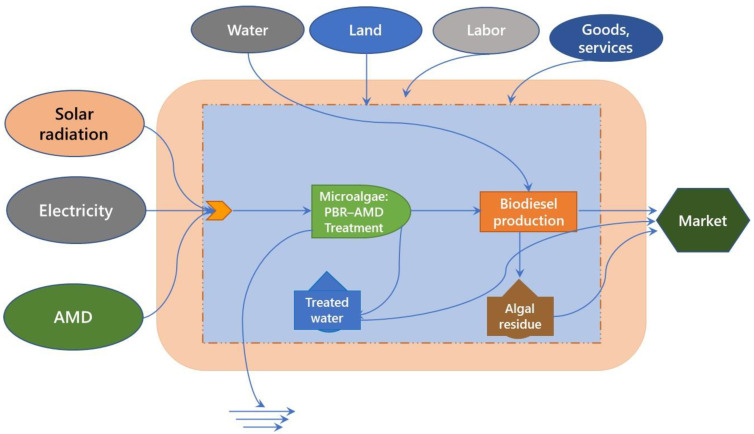
Emergy analysis in AMD treatment system involving immobilized acid-adapted microalgal strains, *Desmodesmus* sp. MAS1 and *Heterochlorella* sp. MAS3.

The emergy flow diagram indicating the details of inputs, output, and internal material flow of acid-adapted microalgal technology for AMD remediation is presented in Figure 1. AMD and water were the major locally available renewable and non-renewable inputs, respectively, for the systems, while biodiesel from the biomass and algal residue were the by-products from the system. The major loss of water due to evaporation in the present system was considered in biodiesel production. da Cruz and Nascimento [29] also reported that water alone accounted for the major energy loss in renewable flow of oil production from microalgal biomass. The results from emergy analysis of the acid-adapted microalgal system for AMD treatment are presented in Table 1. In the present emergy flow, natural renewable inputs (0.43%), non-renewable inputs (0.05%) and imported inputs (>99%) were the major contributors to the overall emergy value of 1010 × 10^15^ sej for AMD treatment (Table 2). Similarly, Winfrey et al. [27] reported the implication of 94 and 99% imported inputs in active and passive systems, respectively, for treating net alkaline mine drainage. In addition, the overall emergy value obtained for acid-adapted microalgal strains was 87–140% lower than that of passive and active treatment systems [27]. This is due to the less consumption of imported materials used in the treatment system despite the lesser renewable input. Maiolo et al. [18] considered geothermal heat flow, tidal energy, wind, wave energy and rain as renewable input flow for production of dried microalgal biomass from *Tetraselmis suecica* and *Tisochrysis lutea* during outdoor cultivation using flat panel photobioreactor. Similarly, natural renewable input accounted for 7 and <0.01% in the passive and active treatment system, respectively [27]. In the present study, solar energy and water were the major renewable and non-renewable inputs used for the microalgal-based AMD treatment in a photobioreactor. The main advantage of the microalgae-based technology is the use of biomass for biodiesel production following in situ transesterification process where the yield of biodiesel is around 10% [25], which is equivalent to 3.65 × 10^17^ sej and 35% of overall emergy value. However, conventional extraction process of biodiesel from microalgal biomass showed that 59.40% of energy could be attributed to the overall emergy value [18]. Furthermore, enhanced hexane consumption and water loss caused by evaporation during extraction process also accounted for >7% of total emergy used by the system as reported recently by Maiolo et al. [18].

### 2.2. Emergy Indices of Acid-Adapted Microalgal System

The data on emergy indices such as EYR, ELR, ESI and per cent renewability for the acid-adapted microalgal system as compared to those for active and passive treatment systems are shown in Table 2. The index, EYR, assesses the system’s overall contribution to the economy [30]. The larger the net benefit to the society, the higher the EYR. Thus, EYR values <1.0 indicate that the emergy yield is less than the emergy invested, indicating that the system is not economically competitive, and if EYR values are >1.0, the emergy yield is greater than the emergy invested, which suggests that the system is economically competitive [34]. The present acid-adapted microalgal system of AMD treatment achieves a high return on each unit of emergy invested because the EYR value is 1.0. Moreover, the emergy value obtained in the present study is less than the passive and active treatment systems by 1.0 and 8%, respectively. This is mainly due to the use of limited imported inputs in the acid-adapted microalgal treatment system as shown in Table 3. On the other hand, ELR is used to determine how much “pressure” the system exerts on the surroundings. The greater the amount of non-renewable energy utilized, the larger the environmental strain would be. ELR values <2 indicate a low environmental impact (or processes that could use large area of a local environment to “dilute the impact”); values between 2 and 10 indicate a moderate environmental impact; and values greater than 10 indicate a relatively concentrated environmental impact [35]. The value of ELR for the present system of AMD treatment is 111, indicating that it has an extreme environmental effect followed by ATS (100) and PTS (13). Despite the use of less acreage of land for PBR construction than the other treatment systems, the ELR value in immobilized acid-adapted microalgal system was much higher. Although more land is used for PTS, the use of solar photovoltaic power results in lower ETR values. Brown and Ulgiati [36] also reported a greater ELR value (263) in the improved sludge treatment process, despite the contribution of higher per cent of renewable input, and suggested that extremely higher ELR might occur from the expenditure of highly concentrated non-renewable energy inputs in a limited local context. However, the ELR value obtained in the present study warrants consideration of adequate renewable inputs to the immobilized microalgal treatment system. If values of ESI are <1.0, it indicates that the items or processes are not long-term sustainable. Medium-term sustainability seems to be defined by an ESI value between 1.0 and 5.0, whilst long-term sustainability is considered if the ESI value is higher than 5.0 [36]. In addition, the per cent renewability observed was higher in PTS, followed by ATS and the acid-adapted microalgal treatment system. The overall per cent reduction in renewability in the present system was 9 and 80% lesser compared to ATS and PTS, respectively.

### 2.3. Carbon Foot Printing of Acid-Adapted Microalgal System

**Figure 2:**
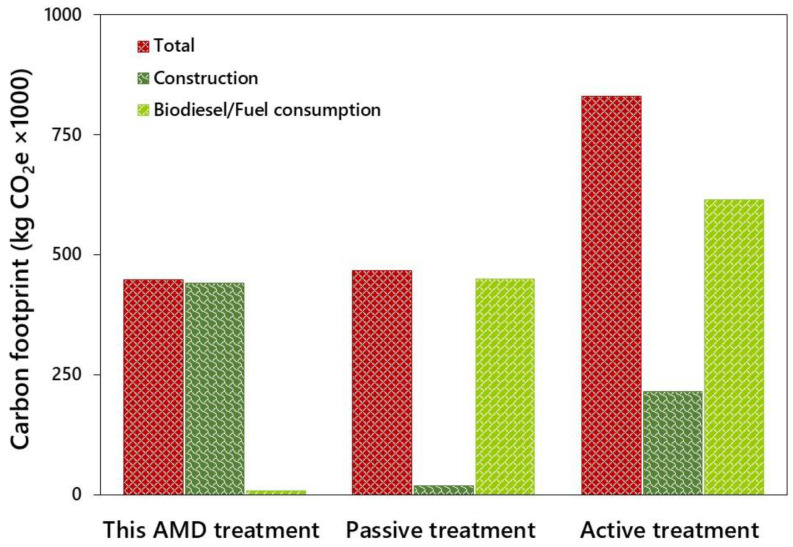
A comparison of carbon footprint - this AMD treatment (immobilized microalgal system), passive treatment [27], and active treatment [27] of AMD.

Based on materials and fuel used during the treatment process, acid-adapted microalgal system emitted less CO_2_, accounting for 4 and 85% reduction compared to that of ATS and PTS, respectively (Figure 2). The significant source of CO_2_ in the carbon footprint in the present study is the construction materials (98%) rather than fuel consumption. However, fuel consumption, in terms of CO_2_ emission, was significant in other systems accounting for 96 and 74% in PST and AST, respectively. Lehtoranta et al. [37] reported that the carbon footprint of small ATS, such as batch reactors and fluidized beds, was greater than that of PTS. Similarly, Martinez et al. [5] highlighted that the procurement and transport of concrete and steel bars made a higher contribution during the construction phase, while the acquisition of carbonate materials and their dissolution generated higher impacts during the application phase, suggesting that the alternative sources of greener raw materials should be explored as substitutes for materials in passive treatment of AMD. Moreover, the disposal of the commonly used chemicals such as limestone after precipitation of heavy metals also contributes to the climate change which is reported to be significant compared to the construction phase [5,7,38]. Hengen et al. [17] demonstrated that ATS using lime slaking had the greatest LCA impacts, while passive treatment approaches had consistently less impacts, except for one PTS with a purchased energy scenario. A 50% reduction in transportation distances resulted in all the scenarios. We also performed the Scope 1 emissions (which are direct emissions) and Scope 2 (indirect emissions due to electrical power purchased from the grid) GHG emissions based on Australia’ s National Greenhouse Energy Reporting (NGER) for the present analysis. In all the treatment processes, the major contributor for the Scope 1 and Scope 2 emission were the electricity and diesel for the acid-adapted microalgal treatment systems, whereas diesel and gasoline were the contributors for both ATS and PTS (Table 3). The total energy consumed was more in ATS and PTS, with majority of the emissions contributed by Scope 1 (diesel and gasoline) while Scope 2 (purchased electricity) alone contributed to the acid-adapted microalgal treatment system. Such a low emissions, based on NGER Scope 1, from the acid-adapted technology are associated with the use of less diesel in transportation of construction materials. However, Scope 2 emissions were prevalent in acid-adapted microalgal systems due to the purchase of electricity for pumping of AMD effluent to the reactor, whereas the conventional systems employ a photovoltaic panel for sourcing electricity for the treatment process, as indicated in Table 3. Additionally, both PTS and ATS contributed N_2_O emissions, with a 100-fold increase by diesel compared to the acid-adapted algal AMD treatment process (Table 3). On the contrary, emergy analysis indicated that construction material influenced CO_2_ emission more significantly than diesel consumption, which is consistent with the results reported by Winfrey et al. [27]. This is because emergy analysis considers the resource use including both renewable and non-renewable inputs rather than construction and diesel consumption that result in less disparity between the systems than carbon footprint analysis [38]. 

## 3. Materials and Methods

### 3.1. Emergy Accounting 

Following the principles of Odum [30], the sustainability of the acid-adapted microalgal technology proposed for acid mine drainage treatment was evaluated via emergy analysis. Emergy is defined as “the quantity of energy (particularly solar emergy) used to develop a resource, both directly and indirectly” [30]. Emergy measures the worth of resources, products, and services in a single unit of energy called solar emergy, which is measured in solar emjoules (sej). All system inputs such as energy, materials, and services are transformed to energy units using a conversion factor termed “transformity” throughout the analysis. The efficiency index is defined as the amount of solar energy necessary to deliver a joule of a product or service (sej/J). For example, the relationship between emergy of a biomass ($Em_{i}$) and its energy content ($E_{i}$) is given by transformity ($Tr_{i}$) as shown in the equation:

Tri=EmiEi

Since solar emergy is the starting point for all other emergy calculations, the transformity of solar energy is set to unity [39]. If the transformity is greater, more environmental assistance is needed to make a product unit accessible [40]. Transformity calculations, in general, have inherent uncertainty in them due to their sensitivity [30]. To address this, researchers employ different approaches to analyzing transformity uncertainty. Thus, several studies have used emergy analysis to compare resource consumption intensity, trade balance, and sustainable production in a variety of systems. Even the application of emergy to geographical locations has resulted in a unique understanding of the regions’ ecology and economy.

**Figure 3:**
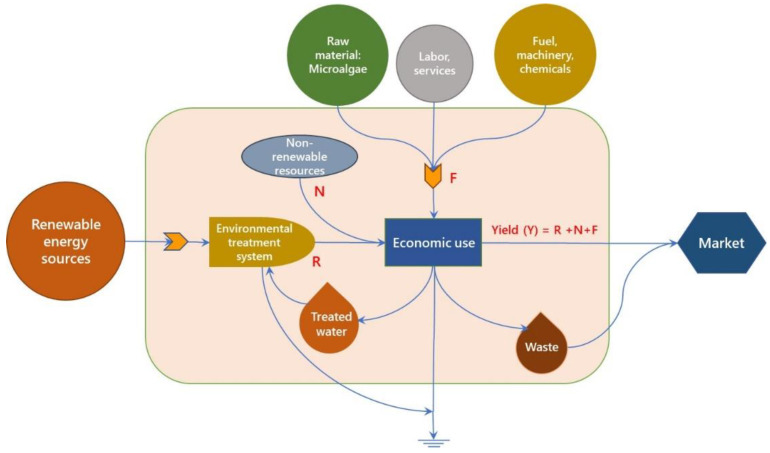
Schematic representation of emergy flow accounting for local renewable energy inputs (R), local non-renewable inputs (N), and purchased inputs from outside the system (F).

Overall, emergy analysis is conducted in a series of easy procedures that include designing a system input and output flow diagram, noting the emergy flows of each item, and computing the emergy indicators. Traditional emergy analysis provides metrics that are ideal for analyzing the system’s ecological and economic prospects to determine long-term growth of the process. Figure 3 depicts the emergy flow diagram, where “R” denotes a renewable resource found in nature, “N” denotes non-renewable energy input, “F” denotes bought non-renewable energy input from the socioeconomic system, and “Y” is emergy production of the process. 

### 3.2. Net Emergy and Emergy Yield Ratio 

The concept of net emergy has been defined as “the emergy yield minus feedback input of a process” [30] and emergy yield ratio (EYR) is “the ratio of the amount of emergy produced (Y) to the emergy purchased from outside the system of society (F)”. The greater the fraction of locally available energy sources (R + N) which are used by means of the investment F, the higher the value of this indicator. EYR helps in predicting the emergy efficiency and economic competitiveness of a local resource based on purchased inputs. 

### 3.3. Environmental Loading Ratio

Environmental loading ratio (ELR) is the sum of non-renewable resource (N) and purchased emergy (F) divided by renewable resource emergy (R). This expresses the environmental services used by the system. When a high value of local renewable resources is used, then ELR decreases, thus indicating a small environmental stress. On the contrary, when a high value of local non-renewable resources is used, it results in an increase in ELR values, thus suggesting a greater environmental stress.

### 3.4. Emergy Sustainability Index

The emergy sustainability index (ESI) was calculated by dividing the EYR by the ELR as it is especially useful for comparing different processes. A sustainable process should expand the ESI. Renewability is a relative measure of per cent renewable of a process or proportion of the total emergy required for a process that is derived from renewable sources. 

### 3.5. System Boundary of Acid-Adapted Microalgae in AMD Treatment

In a previous study, the LCA method was used in the AMD treatment process employing immobilized microalgal system and measured the extent of global warming, acidification, eutrophication, cumulative energy demand, and water consumption consequences [25]. These findings showed that acid-adapted microalgal strains in immobilized technology outperformed limestone or hybrid microalgal treatment systems in terms of environmental sustainability. Following emergy analysis, here we evaluated the environmental and economic perspective of the treatment method by assuming treatment facility installed on a hectare (10,000 m^2^) closer to the mine. Treatment efficiency, recovery, and biodiesel were used in the previous study as inputs for the analysis [25]. Other factors such as solar, wind, and geothermal energies were considered for the Newcastle, Australia area. The AMD treatment approach was examined here from the point of entrance into the treatment system through return of the treated water and the algal biomass to produce value-added products. Emergy inputs include renewable resource emergy, non-renewable resource emergy, and social and services feedback resource emergy. Wastewater treatment, production of microalgal biomass and biodiesel, and the application of algal residue to agricultural land were also included in the present study. A comprehensive assumption and calculation of the emergy data are presented in Table S1. Furthermore, the results of the present study were compared with those of (i) an active treatment system that included an aeration tank, a neutralization basin for lime dosing, and a clarifier, (ii) a passive treatment system that included an oxidation pond, two wetlands, vertical flow bioreactors, re-aeration ponds, and horizontal flow limestone beds, and (iii) a common final polishing cell with emergy value as adopted in the study [27].

### 3.6. Carbon Footprint Analysis

To calculate the carbon footprint, carbon dioxide equivalents (CO_2_e) were used [27]. CO_2_, methane (CH_4_), and nitrous oxide (N_2_O) were transformed to CO_2_e based on their respective global warming potential, usually calculated on a mass basis [41]. CH_4_ and N_2_O have global warming potentials of 25 g CH_4_/g CO_2_e and 298 g N_2_O/g CO_2_e, respectively [41]. All the materials and fuels used during construction were multiplied by their corresponding emission factors to calculate the carbon footprint of a system. The emergy analysis of the passive treatment system (PTS) and active treatment system (ATS) used emission factors established in the literature to multiply the construction material inputs and fuel use [42,43,44]. In addition, direct Scope 1 and indirect Scope 2 emissions were calculated based on Australian National Energy Reporting System [45]. For this, the electricity data and diesel consumption were used in appropriate index to generate CO_2_ equivalent values. 

## 4. Conclusions

The present study evaluated the sustainability of immobilized acid-adapted microalgal system for bioremediation of AMD by comparing with ATS and PTS following emergy and carbon footprint analysis. Emergy analysis showed that renewable energy input was extremely low in the case of the microalgal treatment system, although the total emergy value was lower than in other treatment systems. This was consistent with emergy indices, particularly with higher ELR value and lower per cent renewability than for PTS and ATS. The emergy yield ratio was close to 1.0, indicating a high return on each unit of emergy invested from the treatment process. In addition, CFA revealed that CO_2_ emission in microalgal treatment system was reduced by 80 and 5% compared to ATS and PTS. The primary source of CO_2_ is the construction materials for acid-adapted microalgal treatment process and diesel consumption for the other treatment processes. NGER-based analysis also indicated that energy consumption was greater in ATS and PTS than in microalgal technology as energy is derived from biodiesel produced in the latter system. However, Scope 1 emission in acid-adapted microalgal technology was significantly lower than in ATS and PTS, whereas Scope 2 emission was higher in the former system. Overall, the use of immobilized acid-adapted microalgae for AMD remediation is environmentally friendly and the observed sustainability can be improved by incorporating more renewable energy inputs.

## Figures and Tables

**Table 1:** Emergy calculations for microalgae-based AMD treatment and biodiesel production.

Type of Flow	Item of Emergy Flow (Unit)	Unit Value	Transformity (Sej/Unit)	Emergy (Sej/y)	Reference
R	Locally available renewable inputs				
	Solar energy (J)	3.69 × 10^13^	1.00	3.69 × 10^13^	[30]
	AMD inflow (J)	2.35 × 10^9^	3.80 × 10^6^	8.92 × 10^15^	[23]
N	Locally available non-renewable inputs				
	Land use (m^2^)	1.00 × 10^4^	8.67 × 10^10^	8.67 × 10^14^	[31]
	Water for Biodiesel (J)	4.32 × 10^7^	7.30 × 10^6^	3.15 × 10^14^	[32]
F	Imported inputs in AMD treatment				
	Algal biomass— Inoculum (g)	1.58 × 10^9^	3.16 × 10^7^	5.00 × 10^16^	[29]
	PBR − PVC (g)	1.62 × 10^7^	9.09 × 10^9^	1.48 × 10^17^	[18]
	Electricity (J)	3.85 × 10^7^	1.19 × 10^5^	4.58 × 10^12^	[32]
	PBR steel (g)	2.40 × 10^8^	1.80 × 10^9^	4.32 × 10^17^	
	PBR concrete (g)	1.02 × 10^6^	1.09 × 10^9^	1.11 × 10^15^	
	Labor (J)	2.19 × 10^3^	7.44 × 10^6^	1.63 × 10^10^	
Y	Output after treatment				
	Algal wet weight	1.62 × 10^9^	9.07 × 10^7^	1.47 × 10^17^	[32]
	Treated AMD outflow (J)	2.35 × 10^9^	4.99 × 10^6^	1.17 × 10^16^	[33]
F	Imported inputs in Biodiesel production				
	Steel (g)	1.94 × 10^4^	1.80 × 10^9^	3.50 × 10^13^	[32]
	Concrete (g)	1.75 × 10^5^	1.09 × 10^9^	1.91 × 10^14^	
	Electricity (J)	2.31 × 10^9^	1.19 × 10^5^	1.20 × 10^14^	
	Methanol (g)	2.89 × 10^5^	2.28 × 10^8^	6.58 × 10^13^	
	HCl (g)	2.15 × 10^6^	3.64 × 10^9^	7.84 × 10^15^	
	Services ($)	1.09 × 10^5^	2.22 × 10^12^	2.42 × 10^17^	
	Labor (J)	1.53 × 10^10^	7.44 × 10^6^	1.14 × 10^17^	
	Diesel for transportation (J)	4.39 × 10^9^	1.21 × 10^5^	5.31 × 10^14^	
Y	Output after biodiesel production				
	Residue (g)	1.60 × 10^9^	5.22 × 10^8^	8.37 × 10^17^	[32]
	Algal biodiesel (g)	1.62 × 10^7^	1.69 × 10^10^	2.74 × 10^17^	

**Table 2:** Emergy indices for the microalgae-based AMD treatment.

Emergy Index	Unit/Formula	Value		
		This Study	PTS *	ATS *
Natural renewable	(10^15^ sej)	8.96	134	25
Natural non-renewable	(10^15^ sej)	1.18	NA	NA
Imported inputs	(10^15^ sej)	996	1800	2500
Yield	(10^15^ sej)	1270	NA	NA
Total potential energy (Ep)	R + N + F (10^15^ sej)	1010	1900	2500
Transformity	Y/Ep	1.26	NA	NA
EYR	(R + F)/F	1.01	1.08	1.01
ELR	(F + N)/R	111	13	100
ESI	EYR/ELR	0.01	0.08	0.01
% Renewability	R/(F + R) × 100	0.89	6.92	0.99

* Winfrey et al. [27]; PTS = Passive treatment system; ATS = Active treatment system; NA = Not available; EYR = Emergy yield ratio; ELR = Environmental loading ratio; ESI = Emergy sustainability index.

**Table 3:** Direct and indirect greenhouse gas emissions based on Australia’s National Greenhouse Energy Reporting (NGER).

**Process (Transport)**	**Input**	**GHG Emissions (Scope 1)**			**Total Scope 1 Emission**	**Total Energy GJ**
		**CO_2_**	**CH_4_**	**N_2_O**		
This study	0.317 kL diesel oil	1	0	0	1	12
PTS	32.50 kL diesel oil	88	0	1	89	1255
	3.23 kL gasoline	7	0	0	7	110
					96	1365
ATS	47.50 kL diesel oil	128	0	1	129	1834
	6.22 kL gasoline	14	0	0	14	213
					143	2046
**Process (Purchased Electricity)**	**Input**	**Emission Factor**			**Total Scope 2 Emission**	**Total Energy GJ**
		**(NSW, Australia)**				
This study	10.69 kWh	0.81			0	0
	641 kWh				1	2
PTS	–	0.81			–	–
ATS	–	0.81			–	–

PTS = Passive treatment system; ATS = Active treatment system.

## Data Availability

Not applicable.

## Supplementary Materials

The following are available online. Table S1: Assumptions and calculation of emergy data of immobilized acid- adapted treatment system for AMD bioremediation; Table S2: Summary of direct and indirect emissions of the treatment process.
