# Supplementary material for: Sustainability Evaluation of Immobilized Acid-Adapted Microalgal Technology in Acid Mine Drainage Remediation following Emergy and Carbon Footprint Analysis
Source: Molecules. 2022 Feb 2;27(3):1015. doi: 10.3390/molecules27031015 (PMC8839157; doi:10.3390/molecules27031015)
Supplement: Supplementary file 1 [file molecules-27-01015-s001.zip › molecules-1550323-supplementary.pdf]

Supplementary Information

Sustainability evaluation of immobilized acid-adapted microalgal technology in acid mine drainage remediation following emergy and carbon footprint analysis

Kuppan Praveen[1], Sudharsanam Abinandan[1,2], Kadiyala Venkateswarlu[3], Mallavarapu Megharaj[1,2*]

[1]Global Centre for Environmental Remediation (GCER), ATC Building, College of Engineering Science and Environment, University of Newcastle, Callaghan, NSW 2308, Australia

[2]Cooperative Research Centre for Contamination Assessment and Remediation of Environment (CRC CARE), ATC Building, University of Newcastle, Callaghan, NSW 2308 Australia

[3]Formerly Department of Microbiology, Sri Krishnadevaraya University, Anantapuramu 515003, India

**Address for correspondence*:

Prof. Mallavarapu Megharaj
Global Centre for Environmental Remediation (GCER)
Faculty of Science
The University of Newcastle, ATC Building
University Drive, Callaghan, NSW 2308, Australia
Mobile: +61 411126857
E-mail: megh.mallavarapu@newcastle.edu.au

Experimental setup used in the present study

Microalgal treatment system

Industrial scale photobioreactor (PBR) design (2 × 5 × 8 m), as described by Maiolo et al. [1], was adopted in the present study. The reactor consisted of interconnected vertical cylindrical transparent PVC tubes with 10 cm dia, and the entire structure built using stainless steel frames followed by concrete for supporting the base of the structure. Using a circulation pump, AMD (1000 m^3 per day) is circulated into the reactor and aerated using air compressor. With a Solar insolation area of 7500 m^2, microalgal growth in AMD is assumed to be at 433 kg, (for 1000 m^3) based on our previous study [2].

Passive treatment system (PTS)

The land area used to treat mine drainage as per PTS was 2.7 ha as previously described by Winfrey et al. [3]. This system was developed to remediate mine drainage via the use of gravity flow and renewable energy inputs. Air compressors are driven in one reaeration pond by a solar photovoltaic-charged battery and manually in another by a windmill. The design life of the system is projected to be 30 years. Limestone, concrete, timber (for boardwalk supports), geotextile liners, and plumbing and aeration equipment are used in the construction. Vertical flow bioreactors consisted of a mixture of mushroom compost, wood chips, and artificial limestone sand. The organic component of this mix was excluded from the emergy and carbon footprint studies because it was a waste item that did not represent a resource use or carbon emission (i.e., compost manufacturing was believed to be a nett carbon sink). However, the usage of fuel for the transportation of all commodities was reduced.

Active treatment system (ATS)

An ATS, as described by Winfrey et al. [3] that includes an aeration tank, a neutralisation basin for lime dosing, and a clarifier, was adopted in the present study. PVC, concrete, steel, plumbing, and aeration equipment are used in construction. Concrete aeration tanks and neutralisation basins with a capacity of 2,100 L and a wall thickness of 0.3 m were constructed. Clarifiers had a dia of 5.6 m and a wall thickness of 0.3 m. Pumps for influent and high-density sludge return were specified in the design. Aerators and mixers were sized appropriately for aeration tanks and neutralisation basins. Between unit operations, the system was gravity-fed. The design includes a lime hopper with a capacity of 54.4 tonnes and a utility shed.

Table S1. Assumptions and calculation of emergy data related to immobilized acid-adapted microalgal system for AMD bioremediation.

Emergy inputs	Assumptions and calculated values of an input
Solar energy	Solar radiation = (plant surface area) × (annual solar radiation) × (1.0 – albedo).<br>Assuming the treatment plant is located at Newcastle (AUS), the average solar radiation considered was 6.2E+09/yr. Taking insolation area as 7500 m^2 and albedo as 0.30, the unit solar emergy value calculated was 6.20E+0.90*7500*(1.0-0.3) = 3.69E+13.
Land use	Land area for photobioreactor (PBR) facility assumed was 1.0 hectare or 10,000 m^2.
AMD wastewater	Assuming the flow of 1000 m^3 per day, the average annual flow would be 3.65E+05 m^3. When this value was multiplied with the Gibbs free energy value of 6430, the total unit value calculated for AMD was 2.35E+09 J.
Water for biodiesel extraction	Water for biodiesel extraction used was 4.32E+07 J.
Microalgal biomass as inoculum	Microalgal inoculum volume was considered from results of our previous study [2], wherein 4.334 kg of biomass was used for 1.0 m^3 of AMD to obtain an average annual unit value of 1.58E+09 g.
PVC	Polyvinyl chloride transformity value of 9.09E+9sej g^{-1} was taken from Maiolo et al. [1]. Assuming the construction by Silva et al. [4], the total PVC used in the construction was 2.37 kg per m^3 of wastewater. Considering the density as 3.5 kg m^{-1} [5] and 12 years lifetime, the unit value for PVC material was taken as 1.62E+07 g.
Electricity	As per Silva et al. [4], the operation of PBR involves pumps and compressor, where they consume 7300 and 4070 kwh, respectively. Considering this value for AMD treatment, a total of 1260 kwh/m^3 power is required. Considering the current flow of 1000 m^3, and a project life of 12 years, the unit value of electricity was taken as 3.85E+07 J.
Concrete and steel	Assuming 7.8 kg of steel and 0.03 kg of concrete are used for treating 1.0 m^3 of wastewater [4] and a project life of 12 years, the unit values of concrete and steel were calculated to be 1.02E+06 and 2.40E+08 g, respectively.
Labor	Assuming three operators and three engineers to run the plant covering three shifts, the annual unit value of labor was considered as 2.19E+03 J.

After treatment – algal wet weight	From our previous study [2], the treatment of AMD using microalgae was assumed to produce 4.44 kg algal biomass per m^3. The calculated unit value of microalgal wet weight was 1.62E+09 J.
Biodiesel production, steel, concrete, services, and labor	By considering 0.018 g steel per g of biodiesel, and 0.216 g concrete per g biodiesel produced [6], the unit values calculated for steel, concrete, services, and labor were 1.94E+04, 1.74E+05, 1.09E+05 and 1.53E+10, respectively.
Biodiesel production, electricity, methanol, HCl	Electricity used for transesterification process was calculated by multiplying energy demand (Ed) for transesterification per g algal biomass (J/g) and amount of biodiesel per year. Based on the data from our previous study [2], methanol and HCl values were assumed.
Transport	Assuming a single trip distance of 100 km and truck capacity of 26 MT, with fuel consumption at 0.32 km/L of diesel [7] and energy conversion at 41.5E+06 [8], the total unit value calculated for the diesel used for transportation was 4.39E+09.

Table S2. Summary of direct and indirect emissions of the treatment process.

Emission	PTS	ATS	This study
Total scope 1 emission (t CO_2e)	96	143	1
Total scope 2 emission (t CO_2e)	NA	NA	1
Total energy consumed (Gj)	1365	2046	15
Total energy produced (Gj) (algal biodiesel)	NA	NA	643

PTS = Passive treatment system; ATS = Active treatment system; NA: Not available

References

1. Maiolo, S.; Cristiano, S.; Gonella, F.; Pastres, R. Ecological sustainability of aquafeed: an emergy assessment of novel or underexploited ingredients. *J. Clean. Prod.* **2021,** *294,* 126266.
2. Abinandan, S.; Praveen, K.; Subashchandrabose, S.R.; Venkateswarlu, K.; Megharaj, M. Life cycle assessment for the environmental sustainability of the immobilized acid-adapted microalgal technology in iron removal from acid mine drainage. *ACS Sus. Chem. Eng.* **2020**, *8*, 15670–15677.
3. Winfrey, B.; Nairn, R.; Tilley, D.; Strosnider, W. Emergy and carbon footprint analysis of the construction of passive and active treatment systems for net alkaline mine drainage. Mine Water Environ. 2015, 34, 31–41.
4. Silva, A.G.; Carter, R.; Merss, F.L.; Corrêa, D.O.; Vargas, J.V.; Mariano, A.B.; Ordonez, J.C.; Scherer, M.D. Life cycle assessment of biomass production in microalgae compact photobioreactors. *GCB Bioenerg*. **2015**, *7*, 184–194.
5. Geber, U.; Björklund, J. The relationship between ecosystem services and purchased input in Swedish wastewater treatment systems —a case study. *Ecol. Eng.* **2001**, *18*, 39–59.
6. Jarméus, C. Emergy analysis of biodiesel and biogas production from Baltic Sea macro algae. Master of Science Thesis, KTH Royal Institute of technology, Stockholm, Sweden **2013**.
7. Börjesson, P.; Berglund, M. Environmental systems analysis of biogas systems—Part II: The environmental impact of replacing various reference systems. *Biomass Bioenergy* **2007**, *31*, 326–344.
8. Karlsson, S. Sustainable use of Baltic Sea natural resources based on ecological engineering and biogas production, unpublished thesis Kungliga tekniska högskolan. **2009**.
